# Examining and Contextualizing Approaches to Establish Policy Support Organizations – A Critical Interpretive Synthesis

**DOI:** 10.34172/ijhpm.2020.181

**Published:** 2020-09-30

**Authors:** Sultana Al Sabahi, Michael G. Wilson, John N. Lavis, Fadi El-Jardali, Kaelan Moat, Marcela Vélez

**Affiliations:** ^1^Health Policy PhD Program, McMaster University, Hamilton, ON, Canada.; ^2^McMaster Health Forum, McMaster University, Hamilton, ON, Canada.; ^3^Department of Health Evidence and Impact, McMaster University, Hamilton, ON, Canada.; ^4^Centre for Health Economics and Policy Analysis, McMaster University, Hamilton, ON, Canada.; ^5^Department of Political Science, McMaster University, Hamilton, ON, Canada.; ^6^Africa Centre for Evidence, University of Johannesburg, Johannesburg, South Africa.; ^7^Knowledge to Policy Center, American University of Beirut, Beirut, Lebanon.; ^8^Universidad de Antioquia, Medellin, Colombia.

**Keywords:** Knowledge Translation, Policy Support Organization, Evidence-Informed Policy-Making

## Abstract

**Background:** In response to worldwide calls for the need to support evidence-informed policy-making (EIPM), more countries are increasingly interested in enhancing their efforts to use research to inform policy-making. In order to inform the efforts of those asked to lead the support of EIPM, our aim is to develop a conceptual framework to guide the process of establishing a policy support organization (PSO).

**Methods: **We conducted a critical interpretive synthesis (CIS). We conducted a two steps literature review. In the second step, we systematically searched OVID EMBASE, PsychInfo, HealthStar, CINAHL, Web of Science, Social Science Abstract, Health Systems Evidence, and ProQuest Dissertations and Theses Global databases for documents reporting the establishment of PSOs and the contextual factors influencing the process of establishing these organizations. We assessed the eligibility of the retrieved articles and synthesized the findings iteratively.

**Results:** We included 52 documents in the synthesis. Our findings suggest that a PSO establishment process has four interconnected stages: awareness, development, assessment, and maturation. The process of establishing a PSO is iterative and influenced by political, research and health systems contextual factors, which determine the availability of the resources and the trust between researchers and policy-makers. The contextual factors have an impact on each other, and the challenges that arise from one factor can be mitigated by other factors.

**Conclusion: **For those interested in establishing a PSO, our framework provides a road map for identifying the most appropriate starting point and the factors that might influence the establishment process. Leaders of such PSOs can use our findings to expand or refine their scope of work. Given that this framework focuses only on PSOs in the health sector, an important next step for research would be to include other sectors from social systems and identify any additional insight that can enhance our framework.

## Background

 There are consistent calls worldwide to ensure the use of research evidence in health policy-making to strengthen health systems and to address the ‘quadruple aim’ of enhancing patient experiences, improving population health with manageable per capita costs and positive provider experiences.^1-3^ Using research evidence can help policy-makers make more rational, systematic, and transparent decisions throughout the policy development cycle, namely agenda setting, policy formulation, policy implementation, and policy evaluation.^2,4-7^ More precisely, evidence can help in clarifying a problem, framing viable options to address a problem, identifying implementation considerations (ie, the potential barriers and windows of opportunities) and developing monitoring and evaluation plans that enable rapid-cycle improvements over time to implemented polices.^4-6^ Despite the advantages of evidence-informed policy-making (EIPM) and the worldwide call to increase the use of research evidence in policy-making, several barriers constrain the use of research evidence in health-system policy-making processes.^8-11^

 One of the challenges that policy-makers face in using evidence is related to the complex nature of the policy process in which research evidence is only one factor among many that needs to be considered in policy decisions.^8-12^ Other factors include institutional constraints, interest-group pressure, values, and “external” events (eg, economic recessions).^8-11^ Another challenge is that research evidence can be difficult to use and the results are often packaged and presented in a way that appears to be unhelpful for the types of decisions policy-makers face.^11,13-15^ Studies have also found mutual mistrust between researchers and policy-makers, and that policy-makers can have a tendency to place little value on research evidence as an input into policy decisions.^9,14^ In addition, timely access to high-quality and relevant research and the organizational characteristics of the policy-related organizations further influence the use of research evidence.^16-18^

 There are different mechanisms to overcoming these barriers to support the use of research evidence in policy-making.^12,15,19-21^ For example, knowledge producers or an intermediary group can make it easier for policy-makers to find research evidence when a demand for it arises (eg, through easy-to-search one-stop-shops or clearinghouses for relevant and high-quality research evidence) and to use the evidence they find (eg, by preparing user-friendly summaries of policy-relevant systematic reviews).^22^ Knowledge users can support the processes and structures that are needed to facilitate the demand for evidence from policy-makers (eg, by creating routine processes in the policy development process to use key sources to find and use research evidence).^22^ Furthermore, many have highlighted the importance of mechanisms that support knowledge producers (ie, researchers and academics) and knowledge users (ie, managers and policy-makers) to work more closely together.^23,24^ For example, knowledge producers and users can have meaningful partnerships that enable them to jointly ask and answer relevant policy questions, such as by convening stakeholder dialogues where policy-makers, stakeholders, and researchers can combine the best-available evidence with their collective insights to spark action to address pressing policy challenges.^22^ Lastly, such approaches from knowledge producers and users can be integrated and embedded in a knowledge translation platform (KTP) to enable more comprehensive efforts to support EIPM.^22,25^

 A KTP is a form of organized effort (ie, organization or network) to bring research and policy communities together. A KTP can have five primary objectives: (*i*) identifying policy needs and priorities; (*ii*) harvesting local evidence and experience (eg, by building a database of locally produced evidence) and harmonizing it with global knowledge to guide policy development and implementation; (*iii*) brokering among policy-makers and researchers on key issues; (*iv*) packaging evidence for target audiences; and (*v*) strengthening the capacities of researchers to generate better evidence, and of policy-makers to better find and use research evidence.^26^ This type of approach has been operationalized by the World Health Organization (WHO) through the Evidence-Informed Policy Networks, which has the goal of supporting the process of translating research evidence into policy and action in a number of low- and middle-income countries.^25^ EIPM initiatives have different forms and have been called different names,^9,27^ and recently, some studies discussed the organizational factors that influence the utilization of research evidence by policy-makers and the importance of institutionalizing EIPM efforts.^12,17,18^ Although there is a tendency to assign the EIPM efforts to a particular organization, little is known about the process of establishing such organizations.

 In this synthesis we are calling the organized effort (this could be a department, a unit, a forum, a network, an organization, or an initiative either external from government or embedded within government) to support EIPM a policy support organization (PSO), and our aim is to develop a conceptual framework that can guide the process of establishing a PSO or similar entities. As more countries are increasingly interested in enhancing their efforts to use research to inform policy-making, a trend has emerged where a particular group, initiative, department, network, or organization is asked to lead efforts to support EIPM at the system level. However, despite the increased interest in establishing PSOs we are not aware of an existing synthesis of evidence or a conceptual framework that focuses on the different approaches used to establish such organizations in different contexts, which takes into consideration the establishment process, the organizational attributes or features, and the contextual factors that affect the process.

 This synthesis seeks to address this gap by undertaking a critical interpretive synthesis (CIS) to develop a conceptual framework for establishing a PSO.

## Methods

###  Design 

 We conducted a CIS, which is a synthesis approach designed to analyze a broad range of relevant sources and use analytical outputs to develop a conceptual framework.^28-31^ CIS is a particular form of systematic review that draws both on traditions of qualitative research inquiry and on systematic review methodology.^32^ A CIS is best suited to study an emerging phenomenon that is currently difficult to define,^33,34^ which is the case with processes for establishing PSOs. In conventional systematic reviews, the researcher formulates a precise question that is tightly focused, allowing for pre-identification of the review parameters and the selection criteria. Developing a narrow research question is useful where the phenomenon of interest and relevant populations, interventions, and outcomes are all well-specified.^35^ In contrast, CIS methodology allows for flexibility to draw from a wide range of relevant sources and is not constrained by including only pre-specified designs or quality of documents. Instead, the relevance of the article is the most critical judgment for article inclusion.^28,30^ An additional strength of the CIS approach is that it allows researchers to formulate an initial compass question that can be further iteratively modified and defined as the synthesis progresses.^30,36^

 Our initial compass question “what are the key features (infrastructure, activities, outputs, outcomes and impacts) of the organization, initiative or network that support EIPM by clarifying problems, selecting options, and identifying implementation considerations, and how are these key features related to political, health system and research system contexts, particularly as they help to support evidence-informed health policy-making?” This question was iteratively refined as the literature search, review, and analysis proceeded. The finalized compass question was as follows: what is the process of developing a PSO, what are the main features/ attributes of PSOs, and what are the contextual factors influencing this process?

 The overall goal of both questions is to understand the process of establishing a PSO. However, the initial question aims to identify patterns in the organizations’ features and approach of establishment with particular contextual factors or setting. For instance, we were aiming to understand whether PSOs in low- and middle-income countries have different features or approaches than PSOs in high-income countries.

 During the data extraction and synthesis, we realized that this goal could not be achieved due to lack of evidence. Therefore, we modified the research question to focus mainly on understanding the process of establishing a PSO. Modifying the compass question is a unique feature of CIS, where the compass question is iteratively developed throughout the research process because the phenomenon under consideration is emerging and difficult to define.

###  Eligibility Criteria 

 We included documents that focused on one or more PSOs (ie, organizations, initiatives, and networks that support evidence-informed health policy-making by clarifying problems, selecting options, and identifying implementation considerations), as well as documents that focus on organizational attributes and contexts. We excluded documents that focused on clinical practice or clinical practice guidelines, public health practice, health technology assessments, and knowledge translation (KT) of decision-making in other sectors (not health). We also excluded documents that did not have an explicit description of a PSO (eg, discussing KT or EIPM in general without pointing to a particular organization(s)). We restricted our search to English publications only, and no regional restrictions applied.

###  Search Strategy 

 To identify relevant literature, we used a two-step search strategy that was conducted in October 2018. First, we conducted a preliminary search in Google Scholar for potentially relevant documents in addition to screening documents the research team were familiar with and had highlighted as relevant to the topic. This search identified 38 documents that highlighted 56 different PSOs. Twenty-eight PSOs were excluded after consulting the research team because they did not fit within the inclusion criteria, mainly because they focus on clinical practice guidelines and health technology assessments instead of the health system policies. Of the remaining 28 PSOs, 22 were found to have a web page. The websites of relevant organizations were scanned for descriptors used to discuss PSOs. These descriptors were then utilized to inform the second step, which was the development of a comprehensive database search strategy.

 Using these descriptors, we worked with a librarian at the McMaster Health Sciences Library to develop an explicit and structured approach to search the indexed and grey literature using nine databases to cover the broadest possible spectrum of articles related to the establishment of a PSO. The search strategy used a combination of Medical Subject Heading (MeSH) terms (eg, decision-making, policy-making) and keywords developed for OVID MEDLINE and adapted for other databases as necessary. The search terms were derived by identifying synonyms for five domains relevant to the compass question (the input or what the organization uses, the target or what the organization targets, the connection between the input and the target/what the organizations do, the focus of the organization, and organization descriptor (eg, unit, department, network, organization, forum, platform). The Boolean operators OR were used to combine the MeSH terms and keywords within each concept while AND was used to make the connection across the concepts.

 We developed and piloted ten search strategies to test for their sensitivity. This included testing all synonyms individually for sensitivity, and synonyms that expanded the results to an unmanageable number (eg, 40 000+) were refined by scanning the first three pages of search results. If nothing relevant was found, the synonym was dropped. If some relevant articles were identified within the first three pages of search results, the synonym was included, but limited to title only. The final search strategy for Ovid MEDLINE is summarized in Supplementary file 1 and has been adjusted to further search OVID EMBASE, PsychInfo, HealthStar, CINAHL, Web of Science, Social Science Abstract, Health Systems Evidence, and ProQuest Dissertations and Theses Global. These searches were then supplemented by a hand-search of each included document’s bibliography and reports on the WHO general website to identify any additional relevant literature.

###  Selection of Documents 

 We used referencing software (Endnote version 9) to manage the retrieved documents and to remove duplicates. All documents published up until October 2018 have been considered in our review. To ensure the included documents met the synthesis criteria, two reviewers independently reviewed a randomly generated sample of 20% of the retrieved documents at two stages. First, the principal investigator (PI) reviewed the titles and abstracts of all documents retrieved and classified them as potentially relevant or not relevant (to be excluded), and the second reviewer independently reviewed the title and abstract of 20% of the retrieved documents. The PI conducted a 30-minute meeting with the second reviewer to explain the topic, the inclusion and exclusion criteria, and the strategy to screen the documents. The eligibility criteria were tested by each reviewer independently assessing the first 5% of the search results. Following discussion to reconcile any area of discrepancy, both reviewers then assessed the remaining documents in the sample. Next, the PI reviewed the full text of each document that had been classified as potentially relevant and the second reviewer reviewed 20% of the sample. A Kappa statistic was calculated for the documents reviewed by both reviewers. All discrepancies were resolved by extensive discussion to establish consensus. By reaching a sufficient level of agreement the same inclusion and exclusion criteria were then used to review the remaining 80% of the retrieved documents by the PI.

###  Data Extraction and Analysis 

 We developed and piloted a standardized data extraction form that included data about the documents (the year published, document type, methods employed, countries included and concepts covered), the PSO covered in the paper (organization name and attributes, including leadership, governance, human resources, financial arrangements, linkages, infrastructure, program and services), and contextual factors related to the political, health and research system. The extraction form was designed to conceptually map the process of developing a PSO, the influence of the contextual factors on this process as well as on the organizational attributes. The Cochrane KT framework was used to organize the findings about the program and services, which includes six themes: prioritizing and co-producing evidence syntheses, pushing and supporting implementation, facilitating pull, exchanging knowledge and evidence, improving the EIPM climate, and ensuring sustainable KT processes.^37^

 After reading the included full-text documents and extracting findings using the form, data was synthesized interpretively using the constant comparative analysis approach throughout analysis to ensure that the emerging synthesized constructs are grounded in the data. We started by identifying the common themes and concepts with greater attention given to themes that emerged from multiple documents that helped to understand the process of establishing a PSO and how the contextual factors influenced this process. These themes and concepts were then used to develop conceptual constructs that highlight the main stages for establishing a PSO and the contextual factors that influence this process. Finally, the identified constructs were integrated to produce a synthesized argument (conceptual framework) about the establishment of PSOs in relation to the contextual factors and the organizational attributes. This was done with continuous consultation with other members of the research team who have extensive expertise with supporting policy-makers in identifying conceptual gaps and finalizing conceptual framework. During all stages of data extraction and analysis, the principal investigator (SA) kept a memo to track changes in the compass question and any modifications in identifying documents or synthesizing the results.

## Results

**Figure 1:**
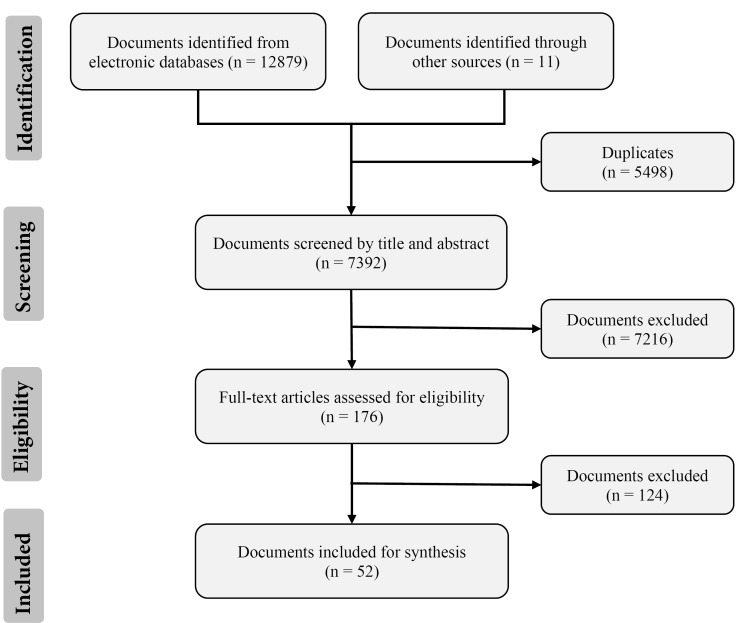
PRISMA Flow Chart for the Review Search Strategy. Abbreviation: PRISMA, Preferred Reporting Items for Systematic Reviews and Meta-Analyses.

 Our search retrieved 12 890 documents. After removing duplicates and screening titles and abstracts, we identified 176 full-text articles for further appraisal, of which 52 documents were eligible for synthesis inclusion (Figure 1). The Kappa statistic on the samples for the first step (ie, screening titles and abstracts of all retrieved articles) and the second step (ie, reviewing the full text of the articles that had been initially classified as potentially relevant) was 0.62 and 0.82, respectively, and both scores reflect substantial agreement between the two reviewers.

 The number of included documents increased over time (2003-2010, n = 8; 2011-onward, n = 44). All WHO regions were represented in the documents, although the organizations predominantly studied were from the African (n = 31) region. Organizations from other regions were less frequently studied, including the Americas (n = 13), South East Asia (n = 10), Europe (n = 7), and Eastern Mediterranean and the Western Pacific (n = 6). Documents also focused on a mix of low-income (n = 27), middle-income (n = 30) and high-income countries (n = 17) (note that several documents focused on countries from more than one income classification). Most of the documents (n = 41) were journal articles, with the rest being commentaries (n = 5), reports (n = 4), meeting abstracts (n = 1), and media articles (n = 1). Of the 52 included documents, 38 were empirical studies. Of these, many employed at least two data-collection methods (n = 19), with document analysis (mainly unpublished internal document, eg, policies, meetings notes, and archives) being the most common method utilized (n = 28), followed by interviews (n = 20), surveys (n = 10), non-systematic reviews (mainly using published documents) (n = 5) and systematic reviews (n = 3). Of the 83 organizations mentioned in the 52 included documents, the most common settings for PSOs were within government (n = 32) or academic institutions (n = 28), while those situated as independent organizations were less common (n = 15). Slightly less than half of the documents focused PSOs from at least two different settings (n = 22) and about one third of the documents focused on PSOs in more than one country (n = 18).

 On June 24, 2020 we ran the same search strategy (presented in Supplementary file 1) in four databases (ie, Medline, HealthStar, Embase, PsycINFO) to identify the documents released between 2019 and 2020. After removing the duplicates, our search revealed 345 articles out of which three were potentially relevant. However, after reading the full documents, non were eligible for synthesis inclusion.

###  Conceptual Framework for Establishing a PSO 

**Figure 2:**
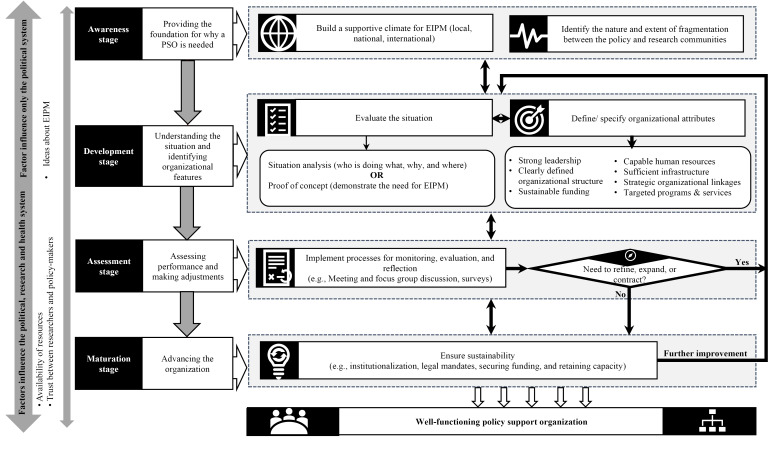
Four Stage Conceptual Framework for the Establishment of a Policy Support Organization. Abbreviations: EIPM, evidence-informed policy-making; PSO, policy support organization.

 We developed a framework that outlines the process of establishing a PSO. Figure 2 presents this framework, which includes four main stages in the establishment process: awareness, development, assessment, and maturation. Each of the stages has unique components as well as connections to the other stages. Although the framework is arranged in a sequential way, it is important to emphasize that the process of establishing a PSO is iterative (this is indicated by the double-headed arrows between the stages) and different organizations may go back and forth between the different stages even after reaching the maturation stage given that the process could be repeated when the organization introduces a new service or program or when an organization goes through an assessment that requires major changes. In addition, some organizations may skip one or more steps depending on what is already in place. For example, if the concept of EIPM is well-established, less work will be needed in the awareness stage, while others might skip the assessment stage when there is not enough capacity to do the assessment. Furthermore, as highlighted in the far left in Figure 2, the process of establishing a PSO is influenced by contextual factors that are related to the political, health and research system. The following sections provide a description of the components and features of each stage, the corresponding contextual factors, and the link between the stages.

####  Awareness Stage – Providing a Foundation for Why a PSO Is Needed

 The awareness stage provides the foundation for establishing a PSO by identifying the motivation that would push the idea of establishing a PSO forward. Two main motivations were identified for this purpose (see Table 1). The first motivation is having a supportive climate for EIPM which is needed to build awareness among policy-makers and researchers about how evidence can inform policy-making.^38-47^ The second motivation is identifying the fragmentation between policy and research communities and the need to address it.^46,48^ A supportive climate or an identified and an agreed upon need to bridge the gap between policy and research communities can each individually drive the need to establish a PSO, as well as act synergistically to provide a stronger case. The level of awareness built from this synergy subsequently affects the development stage. For example, countries that do not have widespread awareness may need to initially focus on developing and implementing programs that help to further build awareness while also supporting EIPM. These programs might include capacity building workshops, priority setting processes, and opportunities for exchange between policy-makers, stakeholders and researchers that create opportunities to help bring the policy and research communities together.

**Table 1:** Description of the Awareness Stage and Contextual Factors Relevant for Establishing a Policy Support Organization

**Description of the Stage**	**Contextual Factors Acting as Mechanisms of Influence on Stages of Establishment **	**Link to the Other Stages**
This stage has two main features that motivate the establishment of a PSO; availability of supportive climate and identifying the fragmentation in the system. • Having a **supportive climate** at the national and/or global level is an essential driver in starting to implement a PSO.^38-47^ • Identifying the problem of **fragmentation and poor connection** between the policy and research community as well as realizing the **need for stronger linkages **between evidence and policy are strong motivations to establish a PSO with a focus on EIPM.^46,48^	**Political system – Availability of resources ** • Availability of aligned support from interest groups and policy-makers creates a supportive climate to advocate for establishing a PSO.^19,39,44,50,51^ • Stakeholder conceptualization of the length and cost of EIPM processes influences the climate for establishing a PSO,^20^ and therefore efforts to clarify the potential outcome of EIPM can help enable a supportive climate for establishing a PSO. **Political system – Trust between policy-makers and researchers ** • Existence of cordial relationship between research and policy communities increases trust over time and minimizes the fragmentation between the two communities. This is important for maintaining or enhancing relationships between the two communities, and for a supportive climate for establishing a PSO.^51-53^ • A positive view about the value of research by policy-makers creates a supportive culture to establish a PSO, and therefore efforts to address negative or uncertain views about the value of evidence should be the initial focus in creating awareness about the need for a PSO.^20,39,44,52^ **Political system – Ideas about EIPM** • Government involvement in international activities that continually call for KT proposals to funding opportunities can further contribute to a supportive climate to establish a PSO.^46,51^ **Research system – Availability of resources ** • Availability of capacity for finding and using research evidence helps to address the fragmentation between the policy and research communities (eg, by conducting more relevant research) and to foster a climate that is supportive of establishing a PSO (eg, by conducting more activities targeting policy-makers to increase their awareness of EIPM).^39,50,51^ **Research system – Trust between researchers and policy-makers ** • Research evidence needs to be interpreted to help identify its relevance, applicability and credibility for policy development,^42,51^ and, therefore, presenting research findings in a user-friendly format is important for supporting the use of evidence in local contexts. **Health system – Availability of resources ** • Existence of policy-development and/or planning units within government institutions contribute to framing the expectation of informing policy using evidence.^52,54,55^ **Health system – Trust between researchers and policy-makers ** • Establishing a PSO in a health system that is highly reliant on external funding (eg, donors or external organizations) is more challenging because it is less trusted and more likely to be fragmented.^55,56^ • In a tiered health system, the competition between private and public sectors might complicate the establishment of a PSO, and therefore it is important to start with identifying common priorities to minimize the fragmentation and increase the trust between the policy and research communities.^52,56,57^	**Stage 2 – Development ** ***Targeted programs and services*** • Increasing awareness is one of the ongoing activities of a PSO to create a supportive climate for any new service the organization may provide.^38,42,44,52,58^ • Building the capacity of policy-makers for EIPM further contributes to building a supportive culture for EIPM.^38,41,49,51,59-62^ ***Clearly defined organizational structure *** • A governance approach that involves researchers and policy-makers can address fragmentation over time.^26,40,46,51,59,63^ ***Strategic organizational linkage*** • Collaboration across research and policy-maker communities improves the contact between them.^26,39,40,42,51,52,59-61,64-67^ ***Evaluating the situation *** • Conducting situation analyses is an important process to convene various stakeholders in order to increase their awareness about the need to further engage and invest in EIPM.^26,38,40,41,43,49^

Abbreviations: EPIM, evidence-informed policy-making; PSO, policy support organization; KT, knowledge translation.

####  Development stage – Understanding the Situation and Specifying the Organization’s Attributes 

 The development stage is the actual implementation of a PSO. This stage is the first point where KT activities start to be more organized and attributed to a specific organization (eg, department, unit, forum, or network). This stage involves identifying the organization’s features by first understanding the situation and then specifying the organization’s attributes (see Table 2). At the early stage of establishing a PSO, different approaches can be used to either understand who is doing what, why and where (ie, situation analysis approach),^26,38,40,41,43,49^ or to provide a proof of concept for efforts to support EIPM among research and policy communities.^40,49^ While a situation analysis approach is better for assessing the relationship between the research and policy communities,^26,38,40,41,43,49^ a proof of concept approach is critical for demonstrating the potential benefit of establishing the organization.^40,49^ These two approaches might play a different role in different countries and they can complement each other. For example, a situation analysis can be used to identify the niche, threats, and opportunities for a newly proposed organization in countries where other organizations that support EIPM in different ways already exist (eg, health technology assessment units that provide decision support for whether to provide funding for specific programs, services and drugs, but not about the system arrangements that are needed to get them to those in need).^26,38,40,41,43,49^ In addition, the same approach can be used to understand policy-makers’ priorities and identify the potential collaborators in countries where EIPM initiatives are dispatched.^40,41^ In contrast, a proof of concept can be helpful in providing direct evidence about or experience with how a PSO can play a critical role in achieving these priorities.^40,49^

**Table 2.** Description of the development stage and contextual factors relevant for establishing a Policy Support Organization

 Once the organization understands the situation, the next step is to identify its attributes, which (as we outline in Table 2) include defining: (*i*) strong leadership^26,40,46,48,54,58-60,63,68-70^; (*ii*) clearly defined organizational structure^26,40,43,46,48,51,52,59,63,64,67,71^; (*iii*) sustainable financial arrangements^39,47,52,59,64,71^; (*iv*) capable human resources^19,21,26,51,52,56,58,62,64,66,72,73^; (*v*) sufficient infrastructure^21,39,41,42,55,56,58,66-68,74,75^; (*vi*) strategic organizational linkages^21,26,39,40,42,49-52,56,59-61,64-67,69,76,77^; and (*vii*) targeted programs and services.^26,39-43,47,48,50,52-54,59-61,65-67,69,70,73,77-80^ In specifying these attributes, it is important to consider them in the frame of the PSO working as a platform that brings together policy-makers, stakeholders, and researchers to support EIPM. Such partnership building and co-creation can underpin all of the attributes of the PSO to maximize the organization’s ability to support EIPM. For example, the leader of the organization should be of high credibility, skills, and expertise in both the research and policy communities.^26,40,46,48,54,58-60,63,68-70^ At the same time, processes for selecting what programs and services to offer may consider involving policy-makers and researchers, especially since this stage will shape subsequent stages in the organization’s evolution, particularly its sustainability.

**Table 2:** Description of the development stage and contextual factors relevant for establishing a Policy Support Organization

**Description of the Stage**	**Contextual Factors Acting as Mechanisms of Influence on Stages of Establishment **	**Link to the Other Stages**
The two main features of this stage are understanding the existing context for establishing a PSO and defining the organizational attributes. ***• Understanding the existing context for establishing a PSO: *** Some organizations ■ Conduct a **situation analysis** before establishing a PSO to understand who is doing what, why, and where^26,38,40,41,43,49^; ■ Other organizations use **a proof of concept approach** by applying some of the proposed KT activities to demonstrate the need for a PSO, thereby garnering support for EIPM.^40,49^ ***• Specifying organizational attributes:*** During this stage the PSO defines seven core attributes ***1. Strong leadership: *** PSO leadership should ■ Have **high credibility** among both policy-makers and researchers to facilitate linkage and build trust; ■ Have **skills and expertise** in both research and policy-making; and ■ Be **institutionalized **to avoid organizational collapse if/when the key people leave.^26,40,46,48,54,58-60,63,68-70^ ***2. Clearly defined organizational structure *** **■ Governance structure **should include a multi-disciplinary, multi-sectoral team to enhance transparency and independence.^26,40,43,46,48,51,52,59,63,64,67,71^ **■ Legal frame and mandates **shouldclearly define the PSO roles and responsibilities to avoid duplication of effort, maximize productivity, and increase the organization access to resources.^43,47,51,63,72^ **■ Location/ ownership/ hosting organization **of thePSO can either be within a governmental or academic institution, or stand on its own.^19-21,38-41,44-48,50-64,66,69-72,74-76,78-84^ ***3. Sustainable funding *** ■ The **funding source **for a PSO might come from international organizations, donors, government, project-based funding from a research funder or another stakeholder group, endowments, or other sources.^26,39-41,44,48,50-52,54,56,59,63-65,71,72,84,85^ **■ Lack of sustainable funding** can slow the development process, and PSOs may have to change hostinstitutions and/or significantly rely on contracts at the end of a donor funding cycle.^39,47,52,59,64,71^ ***4. Capable human resources *** **■ Staffing/hiring: **PSOs need a multidisciplinary team with different areas of content and methodological expertise, and external consultants might be involved to fill some gaps.^19,21,26,51,52,56,58,62,64,66,72,73^ **■ Capacity building: **All PSOs need to continually build and strengthen the capacities of researchers to generate better evidence, and of policy-makers to better enable them to find and use research evidence.^19,26,41,66^ **■ Rewarding: **PSOs oftensuffer from staff turnover due to the low salaries, high workload, and job insecurity, which can be avoided by providing incentives.^26,52,64,65,68,70^ ***5. Sufficient infrastructure *** **■ Facilities: **Institutional infrastructure (offices, equipment, meeting space) influences the practice norms and expectations, and opportunities for skills development and application.^42^ **■ Technology: **A PSO needs technology to function adequately, which includes; personal computers, a functional internet connection, and access to databases (eg, for identifying research evidence).^21,39,41,42,55,56,58,66-68,74,75^ ***6. Strategic organizational linkage *** ■ PSOs tend to build connections with local, national, and international organizations for the purpose of building capacities, pooling resources, enhancing trust between researchers and policy-makers, and conducting joint research and KT activities.^21,26,39,40,42,49-52,56,59-61,64-67,69,76,77^ ***7. Targeted programs and services*** **■ Improving climate/building demand** ■ PSOs continually increase awareness and build demands for their activities and products to improve the climate for EIPM.^38,42,44,52,58^ **■ Prioritization and co-production** ■ Many PSOs embed priority-setting exercises and co-production of relevant research as an essential part of their work.^20,39,48,62,77-79,81^ **■ Packaging and disseminating evidence and support for implementation: **PSOs support the uptake of evidence by disseminating research finding (eg, seminars, media, meetings, publications, and conference) and packaging research in formats that suit users’ needs such as; systematic review, tailored summary, and policy briefs.^26,39-43,47,48,50,52-54,59-61,65-67,69,70,73,77-80^ **■ Facilitating user ‘pull’ for research evidence **by: 1) building the capacity of target users; 2) providing a rapid response service; and 3) administering online clearinghouses or one-stop shops for evidence.^20,21,26,38-44,48,49,52-54,60,62-64,66,70,75-79,84,85^ **■ Exchange: **PSOs conduct deliberative dialogues to exchange ideas with partners, learn about their evidence needs, identify tacit knowledge and actions that can be taken by different groups to address health-system issues, and contextualize global evidence.^26,39,40,43,50,52-54,56,59,60,63,65,66,69,70,77-80^ **■ Sustainable KT processes **involvebuilding capacity for different types of functions (eg, preparing evidence briefs, convening policy dialogues and providing a rapid response services), raising funds, and monitoring and evaluating the impact of the PSO’s work on policy change.^20,38-41,43,48,53,60,64,66,78^	**Political system – Availability of resources ** • Anchoring a PSO to a pre-existing institutional structure facilitates its establishment by pooling needed financial and human resources, sharing infrastructure, and helping to foster support from policy-makers, stakeholders and researchers. For these reasons, institutionalizing the PSO within a pre-existing structure is recommended even if initially started as an independent project.^39,51,52,54,60,63,64,70,76^ • The governance approach of the hosting organization that allows for mobilizing funds for program and project implementation can facilitate the establishment of a PSO, but this requires being fully aware of the administrative formalities of the hosting organization.^39,44,50,51,55,56^ • Having appropriate political support from policy-makers and stakeholders facilitates the establishment of PSOs by mobilizing needed resources and resolving any conflicts between the research and policy communities,^19,39,44,50,51^ and this requires processes to identify policy-makers’ interest and any potential resistance to establishing a PSO. **Political system – Trust between researchers and policy-makers ** • Existence of a cordial working relationship between research and policy communities coupled with regular communication facilitates the establishment of a PSO by allowing researchers to understand policy-maker interests and allowing policy-makers to have a trusted contact when they have specific research questions.^50-53^ **Political system – Ideas about EIPM ** • A high level of awareness among target users about the PSO’s programs and services facilitates the establishment of the organization as it increases their interest to support the organization (technically or financially) and/ or to integrate it in their organization in the case of PSOs that have started as a pilot or small project.^39,52^ • Conceptualization of the length and cost of EIPM processes among policy-makers and stakeholders influences their commitment in providing needed supports and resources to establish a PSO^20^ and, therefore, efforts to clarify the potential outcomes of EIPM can enhance the climate for establishing a PSO. **Research system – Availability of resources ** • Having capable human resources that can understand and use research is essential for establishing a PSO, and having the appropriate incentive(s) to attract and retain such skillful capacities is essential for organizational sustainability.^39,50-52,55,56,68,70^ • Availability and diversity of financial resources to conduct research and/or KT activities facilitates PSO establishment and helps to expand organizational scope.^39,46,50,68,70^ This is particularly important for organizations that are not institutionalized and that are heavily dependent on donors and short-term grants (eg, to avoid collapsing/contracting by the end of the donor’s fund). • Availability of relevant, applicable, accessible, and easy to read research and health information can determine the scope of work the organization can do and how fast the work can be accomplished.^39,42,44,50,51,70^ • Existence of a research department and clear mandate to link research to policy facilitates the establishment of PSOs by enhancing the interaction between researchers and policy-makers and/or by building new connections where needed.^46,52,54,55,57^ **Research system – Trust between researchers and policy-makers ** • Having interaction between researchers and policy-makers helps in pooling resources through finding or conducting relevant research and identifying research grants with KT components.^35,38,40^ • Potentially sensitive research findings (eg, in relation to political priorities) might hinder buy-in for establishing a PSO but this can be mitigated by the organization addressing any potential resistance to research findings by engaging in a collaborative tone and clearly highlighting how they can be helpful to informing government priorities.^19,20,50,51^ • The credibility of researchers (and therefore the research they produce) facilitates the establishment of PSOs by strengthening the relationship between researchers and policy-makers, which then improves organizational linkages and ability to pool human resources that can be used to produce and use.^42^ **Health system – Availability of resources ** • Having highly-qualified managers within an MOH facilitates the establishment of PSOs, because such managers are more likely to value research evidence, be willing to use evidence in decision-making, and recognize and support research processes within the MOH that encourages the usage of research during policy development.^42,44,51,54^ **Health system – Trust between researchers and policy-makers ** • Competition between the private and public sector in a tiered/ mixed health system may slow the process of establishing a PSO (eg, by making it harder to pool needed resources and engage all relevant stakeholders),^52,56,57,76^ which lends further support to the need to start the establishment process with identifying common priorities among stakeholders across different sectors	**Stage 1– Awareness ** ***Building a supportive climate for EIPM *** • A proof of concept raises awareness and helps foster a supportive climate for EIPM by demonstrating the practicality and efficiency of EIPM.^40,49^ ***Identifying fragmentation *** • A situation analysis can help identify fragmentation between policy and research communities that needs to be addressed.^26,38,40,41,43,49^ • Fragmentation between the policy and research communities can be addressed through an organizational linkage that provides common ground for regular communication between the two communities to bridge the gaps in the evidence-to-policy process. ^26,39,40,42,51,52,59-61,64-67^ **Stage 3 – Assessment *****Evaluation and reflection *** • After a period of donor funding is completed, organizations need to assess their situation and performance, which represents a good opportunity to make an adjustment in the organization location, sources of funding, and activities.^47,52,64^ **Stage 4 – Maturation ** ***Ensure sustainability*** • Institutionalization of a PSO within a pre-existing institutional structure is an essential factor to ensure its sustainability.^39,41,44,45,47,51,59^ • A legal framework of a PSO that is framed to reduce duplication of effort, maximize productivity and enhance understanding of stakeholder needs is important for the long-term survival of a PSO.^43,47,51^ • Securing stable long-term funds for a PSO through institutionalization in a pre-existing institutional structure is an important factor to ensure PSO sustainability.^39,47,59^ • Identifying an appropriate approach to retain the human resources needed in a PSO (eg, providing financial and/or non-financial incentives) is essential to ensure organizational sustainability.^39,62^

Abbreviations: EPIM, evidence-informed policy-making; PSO, policy support organization; KT, knowledge translation.

####  Assessment Stage – Monitoring and Evaluating the Organization 

 The assessment stage consists of monitoring and evaluating either specific programs and services provided by the PSO and/or its overall performance. As we outline in Table 3, we found three different approaches for monitoring and evaluating PSO activities: convening meetings/focus groups or conducting interviews;^38,76^ conducting surveys;^38,39,41^ and engaging external experts/agencies.^46,62,76^ Among the few documents that discuss assessment, the focus was mainly on evaluating a specific activity and its corresponding product(s). Some organizations conducted an assessment on a regular basis (eg, annually), while others did so at key junctures (eg, at the end of a donor funding cycle or specific project, or after a training workshop). Some documents reported the importance of assessing the impact of a PSO (or similar entities) in health policy and policy-making process.^39,44,55,69^ Others further reported that such entities had an impact on health policies and policy-making process.^58,64,65,70^ However, none of these documents were explicit about what exactly was assessed (or should be assessed) nor were they explicit about how the impact was assessed.

**Table 3:** Description of the Assessment Stage and Contextual Factors Relevant for Establishing Policy Support Organization

**Description of the Stage**	**Contextual Factors Acting as Mechanisms of Influence on Stages of Establishment **	**Link to the Other Stages**
Monitoring and evaluation of a PSO could be conducted either regularly or at key junctures to assess the overall performance of the organization or to assess specific activities and its corresponding product(s). Several approaches to monitoring and evaluation in a PSO were identified, which include: **• Convening meetings/focus groups or conducting interviews** designed to solicit feedback after the initial planning of the service^38,76^; **• Conducting surveys **to evaluate the outcome of PSO activities (eg, quantitative surveys of changes in knowledge, attitudes, and practice among participants in training workshops)^38,39,41^; and **• Engaging external experts/agencies **to evaluate PSO programs and services.^46,62,76^	**Political system – Availability of resources** • Monitoring and evaluation are essential in the process of developing a PSO, and this stage is influenced by the availability of resources, particularly human and financial resources, that can be mobilized to evaluate the organization and identify any needed adjustments,^39,46,50,51,68,70^ and therefore identifying funding resources that are easy to mobilize and utilize is important. • The level of complexity of the administrative formalities of the hosting organization for a PSO might influence the ability to evaluate a PSO as it can make the tracking process harder and more complicated.^51^ It is important to have a clear organizational structure, legal mandate, resources and task descriptions for the organization to be able to efficiently evaluate its performance in a robust way. • The clarity of the hierarchical consultative and decision-making chains within the hosting organization facilitates the assessment of a PSO and enhances the ability to point to specific areas that need to be changed to improve the organization,^55,56^ but this also requires being fully aware of the administrative formalities of the hosting organization. **Political system – Trust between researchers and policy-makers ** • Involvement of researchers in the policy-making process facilitates the assessment of a PSO by identifying their needs and to design, implement and evaluate activities to meet those needs.^52,76^ **Political system – Ideas about EIPM ** • The openness of policy-makers to change (eg, to hear the limitations of the organization as a way to change for the better performance) can facilitate monitoring and evaluation of a PSO.^19,20,39,50,51^ **Research system - Availability of resources ** • External collaboration with research institutions or similar organizations facilitates a neutral assessment processes for a PSO, and the process is stronger if these institutions share similar context or are at least familiar with the local context.^46^ **Research system – Trust between researchers and policy-makers** • No evidence identified **Health system – Availability of resources ** • No evidence identified **Health system – Trust between researchers and policy-makers ** • No evidence identified	***Stage 2 – Development *** ***Sustainable funding*** • After a period of donor funding is completed, organizations need to assess their situation and performance, which represents a good opportunity to make an adjustment in the organization’s location, sources of funding, and activities.^47,52,64^ ***Capable human resources *** • PSOs might hire an external expert/consultant to fill particular gaps such as reviewing a brief or evaluating PSO outcomes in a neutral way.^46,62,76^ ***Strategic organizational linkage *** • Collaboration and networking between PSOs and other organizations (particularly external agencies) might be utilized for the purpose of monitoring and evaluation purpose.^46,62,76^

Abbreviations: EPIM, evidence-informed policy-making; PSO, policy support organization.

####  Maturation Stage – Approaching Sustainability 

 The maturation stage focuses on ensuring long-term sustainability. Four different approaches were identified to attain sustainability: institutionalization of the PSO^39,41,42,44,45,47,51,59,64^; having a clear legal mandate^43,47,51^; having sustainable funding^39,47,59^; and having appropriate capacity (see Table 4).^39,62^ Although no single document addressed all four approaches, the evidence strongly supported that none of these approaches can solely drive the organization towards the maturation stage. Instead, it is clear from the included documents that all of these components are important for an organization’s stability.

**Table 4:** Description of the Maturation Stage and Contextual Factors Relevant for Establishing a Policy Support Organization

**Description of the Stage**	**Contextual Factors Acting as Mechanisms of Influence on Stages of Establishment **	**Link to the Other Stages**
The maturation stage reflects the organization’s stability where it can be considered as sustainable in the long term. The following are some of the features identified as being important for ensuring sustainability: **• Institutionalization **of PSO within a pre-existing institutional structure to facilitate the ability to overcome challenges.^39,41,42,44,45,47,51,59,64^ • Having a formal** legal mandate **(ie, legislation, ministerial order, term of reference) to reduce duplication of effort, maximize productivity and enhance understanding of stakeholder needs.^43,47,51^ • Having a** sustainable source of funding **to reduce the threat of ending some or all of the organization’s activities when one or more sources for external funding stops.^39,47,59^ • Having mechanisms to **retain needed capacities** (eg, providing financial and/or non-financial incentives).^39,62^	**Political system – Availability of resource ** • Anchoring a PSO to a pre-existing institutional structure facilitates its establishment by pooling needed financial and human resources, sharing infrastructure, and helping to foster support from policy-makers, stakeholders and researchers. For these reasons, institutionalizing the PSO within a pre-existing structure is important even if initially started as an independent project.^39,51,52,54,60,63,64,70,76^ However, the impact of this is influenced by the strength of the anchored organization’s infrastructure, governance, and ability to mobilize the resources to fund and implement programs and projects.^39,44,50,55^ • To ensure the sustainability of PSO, the payment scheme should be attractive enough to retain staff.^39,50-52,68,70^ • The conceptualization of the length and cost of EIPM processes by policy-makers and stakeholders influences their commitment to providing needed supports and resources in the long-term,^20^ which makes it important to demonstrate the potential effectiveness of the PSO in improving resource allocation and other aspects of providing health services. **Political system – Trust between researchers and policy-makers ** • Trust between policy-makers and researchers increases the commitment and support of policy-makers from within the government, which influences the PSO sustainability because there support increases the likelihood of the organization being institutionalized and gaining support from other local and international organizations.^19,39,44,50,51^ **Political system – Ideas about EIPM** •PSO sustainability is enhanced when policy-makers and stakeholders value the role of research in policy-making, which in turn helps build increased awareness among policy-makers about the services offered.^42,44,51,54^ **Research system – Availability of resources ** • Weak productivity of research (particularly local evidence) due to financial or human resources challenges would influence the sustainability of PSO.^50,68,70^ **Research system – Trust between researchers and policy-makers ** • Maintaining a trusting relationship between policy-makers and researchers is important for PSO sustainability because it forms the foundation of all PSO activities, builds stronger inter-organizational links over time, and ensures credibility and neutrality of the PSO.^43,46,68^ **Health system – Trust between researchers and policy-makers ** • Highly qualified managers with a research background within the MOH is important for sustaining the value placed on using research in decision-making, which can enhance the sustainability of PSO as it supports ongoing demanding for and use of PSO services.^44,54^ **Health system – Availability of resources ** • Having a focus on evidence-to-policy processes within a government’s mandate facilitates PSO sustainability because of the pressure the mandate can create on policy-makers to utilize research and developing evidence-informed policies (and to draw on the PSO’s services in the process).^46^	***Stage 2 – Development *** ***Strong leadership *** • Institutional leadership is important to avoid organizational collapse if/when the key people leave.^42,46,70^ ***Clearly defined organizational structure *** • The PSO governance approach, clarity of its legal mandates, and its location are critical to defining organization sustainability.^43,47,51,63,72^ ***Sustainable funding *** • Lack of sustained funding can slow the development process,^39,59,64,71^ and jeopardize the organization’s sustainability.^47,52,64^ ***Capable human resources *** • Any issues with low salaries, high workload, and job insecurity should be resolved to retain the qualified staff in order to ensure long-term sustainability.^26,52,64,65,68,70^

Abbreviation: PSO, policy support organization.

###  Contextual Factors 

 There are facilitators and barriers that influence the establishment of a PSO at the political, research and health system level. The political level influences the process of establishing a PSO by determining the availability of resources needed to establish a PSO, the trust between researchers and policy-makers, and the ideas about EIPM. On the other hand, the research and health system influence the establishment of a PSO by determining the availability of resources needed to establish a PSO and the trust between researchers and policy-makers. Tables 1-4 highlight the influence of each contextual factor at each stage in the development of a PSO, and we summarize the main points of influence in relation to political, research and health system.

####  Political System Factors

 There was a diffusion of ideas about KT and EIPM at the national and international level through conferences, training workshops, and funds for research projects that have a KT component.^46,52,60,66^ Diffusion of these ideas were reported to play a major role in changing the ideas about EIPM by shifting the beliefs of policy-makers regarding the importance of research in policy-making and creating consciousness about the need for stronger linkages between policy and evidence.^42,46^ Therefore, the diffusion of ideas played a role in increasing awareness and building a supportive climate for establishing a PSO.

 At the same time, policy-making processes, existing institutions (eg, planning and research departments), and existing policies influence the establishment of PSO by creating incentive and resources to establish a PSO.^43,46,47,49,52,60,64,75^ For instance, if existing policy-making practices emphasize the importance of using research and policy-makers have experience and expertise in doing so, these provide an incentive and resources (eg, through a supportive climate for establishing a PSO and with experience and expertise that can be used to begin to operationalize a PSO).^51,60,64^ In addition, government structures that enable and support collaboration (eg, through pre-existing collaborations with research institutions) facilitate the establishment of PSOs by increasing the trust between policy-makers and researchers and promote the availability of resources through collaboration and networking.^39,44,50,51,55,56^

 Having committed interest groups (eg, high-level policy-makers, health professionals, academic institutions, government agencies, and stakeholders) who express support and advocate for EIPM have been found to be an essential facilitator for establishing a PSO.^19,20,39,43,50,51,70,74^ The interest of policy-makers has been found to be particularly influential when managers within government are highly qualified and value the role of research in policy-making.^54^ This appreciation can foster a supportive climate throughout government departments for establishing a PSO.

####  Research System 

 Research systems also influence the establishment of PSOs by influencing the trust between researchers and policy-makers, and by influencing the availability of resources for a PSO. Availability of research infrastructure (eg, access to online databases), capable researchers and funding for local research are important inputs and resources for establishing a PSO.^43,46,65,68^ On the other hand, the establishment of a PSO can be hindered if the research system is primarily shaped by the priorities of funders and researcher with implications of poor or no uptake of research outputs for decision-making.^43,46,65,68,70^ Moreover, a lack of communication between the research and policy communities can lead to the mistrust between the two communities and make policy-makers more skeptical in using the findings or other services provided by researchers (eg, because of a perception that researchers do not understand their priorities).^44,50,51,76^

####  Health System 

 Health system arrangements can facilitate or hinder the establishment of a PSO by influencing the trust between researchers and policy-makers and by influencing the availability of resources for a PSO. Having ongoing collaborations and networking between relevant government departments and researchers enhances the trust between them, which can create a more supportive climate for establishing a PSO.^46,51,53^ In addition, well-established trust between the research and policy communities can facilitate the PSO’s ability to run its programs and services using a collaborative approach, which further enhances trust and the production of relevant outputs that can be used to inform policy. However, maintaining trust and making resources available for a PSO becomes more challenging in a health system that is highly dependent on donors because of the deviation between policy authority (ie, the government) and implementation facilitators (ie, the donors).^56,55^ Because establishing a PSO in a health system that is highly reliant on external funding (eg, donors or external organizations) is more challenging because it is less trusted and more likely to be fragmented. In a situation where a PSO is planned to be embedded within a government organization, the PSO can draw on existing infrastructure and more efficiently mobilize resources (eg, infrastructure, human and financial resources) to establish it and support its functions.^56,39,50^

###  Linkages Between Stages

 As we depict in Figure 2 and across Tables 1-4, the four stages for establishing a PSO are interconnected with the actions that take place in one stage affecting the other stages. Most of the connections are centralized around the development stage given that all actions that take place in the development stage have an impact on the other stages. The development stage contributes to the awareness stage in two main ways. First, understanding the situation helps in identifying the motivations (ie, identifying the fragmentation between research and policy communities or raising awareness) needed to create a supportive climate for establishing a PSO.^26,38,40,41,43,49^ The fragmentation might then be addressed through the PSO’s governance approach that involves policy-makers and researchers,^26,40,46,51,59,63^ and by building collaboration with other organizations to run different activities and programs.^26,39,40,42,51,52,59-61,64-67^ Second, the programs and services offered by a PSO can subsequently enhance the climate for the use of evidence in policy-making (ie, it can create a positive feedback loop).^38,42,44,52,58^

 The development stage is also connected to the assessment stage where some of the organization’s attributes might be adjusted after assessing the organization’s performance.^47,52,64^ At the same time, the way the organization’s attributes are specified (particularly its governance approach, human resources, linkages and financial arrangements), determine the organization’s ability to conduct monitoring and evaluation from which the results can be used to inform the PSO’s continued evolution.^46,47,52,62,64,76^

 The maturation stage is connected to multiple aspects of the development stage, including having strong institutionalized leadership,^42,46,70^ having a clearly defined organizational structure and mandate,^43,47,51,63,72^ identifying sustainable sources of funding,^39,59,64,71^ and incentivizing human resources to avoid staff turn-over.^26,52,64,65,68,70^ The way these aspects are defined at the development stage strongly affects the organization’s ability to sustain itself in the long-term.

## Discussion

###  Principal Findings 

 Based on the large and growing volume of evidence in the field, we developed a conceptual framework to inform the process of establishing a PSO. Our findings suggest that a PSO undergoes four stages on the pathway towards becoming sustainable. The four stages include: (*i*) awareness (providing the foundation for why a PSO is needed); (*ii*) development (the actual implementation of a PSO); (*iii*) assessment (assessing performance and making adjustments); and (*iv*) maturation (advancing the organization towards sustainability and institutionalization). While each stage has its unique features and contributes toward the establishment of a PSO, the entire process is iterative and movements between the stages should be expected.

 Although the four stages are interconnected and the activities that take place in one stage influence the other stages, the development stage is central in the process of establishing a PSO and it is the one stage that has a direct effect on all of the other stages. Despite an organization’s age, some may not go through each of the four stages. However, among the organizations in the literature we identified, the development stage was never skipped, because it is the actual stage of implementing a PSO. In contrast, the awareness stage was bypassed in many countries where a wide-spread awareness about EIPM already existed. Similarly, the assessment stage was skipped when there was insufficient capacity and/or skills to do the work. We also found that assessments of PSO mainly focused on evaluating the processes and outputs of a PSO with little evidence available on evaluating their impact on supporting EIPM. This might be because the policy process is complicated and research is only one factor among many others that influence it, which makes conclusions about impact of a PSO supporting evidence use difficult to determine.^8-11^ Therefore, even if a PSO succeeds in supporting policy-makers with the best available evidence, it would be hard to discern the exact influence of research from the other factors. Regardless, the documents we included still emphasize the importance of evaluation given that it can provide insights about how to strengthen PSO activities through formative evaluations and to document any impacts through summative evaluations either indirectly (eg, by measuring effects on behavioural intentions to use research evidence) or directly (eg, though qualitative studies that gather insights about whether and how PSO activities supported evidence use in efforts to strengthen health systems).^58,64,65,70^

 Moreover, the four stages should not be viewed in isolation from important contextual factors that can hinder or facilitate the establishment of a PSO. Our findings indicated that the process of establishing a PSO is influenced by contextual factors in political, research and health systems, which determine the availability of the resources and the trust between researchers and policy-makers. The political system further determines the importance placed on EIPM which is central to enabling the process of establishing a PSO. The contextual factors have an impact on each other, and the challenges that arise from one factor can be mitigated by the other factors.

###  Findings in Relation to Other Studies 

 To the best of our knowledge, this is the first comprehensive literature review examining and contextualizing approaches to establish PSOs. The ever-growing number of studies undertaken to inform efforts to support EIPM differ in important ways from what we have done here. First, many examined the process for a specific approach to supporting EIPM (eg, rapid response, clearinghouses, policy brief) and demonstrated the steps of conducting that particular approach.^44,63,76,84,86^ However, none have focused on how to assign that particular approach or activity to an organization that is solely focusing on supporting EIPM. In addition, the majority of the documents do not distinguish between type of decisions (eg, clinical, public health and health-systems topics) that are the focus of this work. Second, one document focused on identifying the steps in developing a KTP, but it was^82^: (*i*) not based on a comprehensive literature review; (*ii*) limited to the experiences of European countries; (*iii*) did not identify the specific factors that influence each step; and (*iv*) focused on operationalization and launching rather than the entire process. Third, some work has been done on the process of institutionalization, but it was very specific for units performing one particular service (ie, rapid response service), compared to our focus on an organization that can perform multiple KT activities to support EIPM.^47^

 Lastly, the contextual factors that influence the establishment of a PSO were not well-addressed in the literature which limited our ability in making a better distinction about their impact on the different stages of the framework. While literature is available on the contextual factors that affect KT in general or specific KT approaches or activities,^19-21^ there is less emphasis given to factors affecting the establishment of PSOs. The level of state centralization and democratization, the influence of external donors and organizations in the health system policies, the organization and function of bureaucracies, the infrastructural resources, research and KT funding, the framing of evidence in relation to social norms and values, and quality and quantity of research targeting health system are among the factors that were reported to influence research utilization in general.^87-89^ Some of these factors intersect with our finding as factors that influence the establishment of a PSO. Particularly, the political factors (ie, ideas, interest, and institutions), quality and quantity of health system research, availability of resources (ie, infrastructure, human resources, and financial resources), and the role of funders.

###  Strengths and Limitations

 A key strength of this CIS is that it used systematic and transparent methods, but in a way that allowed for flexibility that enabled rich interpretive analysis to generate the conceptual framework. This highlights another strength, which is that we developed a new conceptual framework for the process of establishing a PSO that fills an important conceptual gap in the literature and that can be used by policy-makers, researchers, and stakeholders from different countries to guide their efforts in establishing PSOs.

 A potential limitation is that we focused our synthesis only on organizations that support policy-makers at the health system level, and not on those that support the production and use of other types of decision supports for policy-making (eg, clinical practice guidelines, health technology assessments, or public health practice). The potential limitation stems from the possibility of having missed relevant insights from these areas. However, we identified a large set of relevant documents (n = 52) that provided substantial insight into an area that is not as far advanced conceptually as these other areas. Given this, the tradeoff between breadth and depth in the more specific domain of PSOs that focus on strengthening health systems was important to advance our thinking in the field. Another potential limitation is that while we attempted to identify whether particular features, activities, or establishment approaches are more common in a particular setting (ie, government, academic, independent) or context (eg, country income level or region), this was not possible for two main reasons. First, most of the studies that included more than one organization presented results in an aggregated format, and this did not allow for an in-depth analysis of linked organizational features and contextual factors. Second, the few studies that presented individual cases either did not provide enough contextual background or they did not explain the role of contextual factors in shaping the organization.

###  Implications for Policy and Research

 We have identified several policy implications for those supporting EIPM based on the results of our CIS. For those interested in establishing a PSO, our framework provides a road map for identifying the most appropriate starting point. It also helps in identifying the factors that might influence the establishment process that varies according to the context in which a PSO is to be established. For example, establishing a PSO in a country where sufficient awareness about EIPM already exists will likely not require much effort invested into the first stage. Instead, in such a context, the focus will be shifted to the second stage for evaluating the situation and identifying the organization’s attributes. Furthermore, our findings can be informative for established PSOs. Leaders of such PSOs can use our findings to expand or refine their scope of work, such as by selecting a new program or service to provide and refining monitoring and evaluation plans to include assessments of the impact of their work (if this was not previously included in the scope of monitoring and evaluation efforts).

 Given that this framework focuses only on PSOs in the health sector, an important next step for research would be to include other sectors from social systems and identify any additional insight that can enhance the framework we have developed. This CIS also identifies that assessment of PSO performance is not well-established and therefore future research should focus on identifying approaches for evaluating the impact of PSOs. One approach to do so could be through a before and after quasi-experimental design with a set of indicators to assess the impact of a PSO in informing policies with the best available evidence and whether informing policies by evidence has any impact in the efficiency of the policy-making process and ultimately on strengthening the health system. For already established PSOs, evaluations of impact could be conducted using qualitative methods (eg, interviewing policy-makers, researchers and stakeholders about whether and how programs and services offered by the PSO had an impact on evidence use in policy-making) or conducting theory-informed multiple case studies with clearly defined indicators and with a sampling strategy that would ensure that counterfactual cases are included in order to provide insights about the organization’s impact.

## Conclusion

 This CIS developed a conceptual framework for establishing PSOs and in so doing makes an important contribution to the literature related to supporting EIPM. The framework captures the main features in the process of establishing a PSO, the main organizational attributes that have to be specified during the process, and the contextual factors that might affect this process. The four stages identified in this framework should be carefully considered in relation to the country-specific needs and readiness in adopting an EIPM approach to know what is required in each stage and to know which stage would be the best starting point given local contexts.

## Ethical issues

 Not applicable.

## Competing interests

 Authors declare that they have no competing interests.

## Authors’ contributions

 SAS was responsible for the conception and design, collection of data, analysis and interpretation of data, drafting and reviewing the manuscript. MGW contributed to the conception and design, analysis and interpretation, drafting and reviewing the manuscript. MGW was also responsible for supervising this work. JNL, FEJ and KM provided critical revisions of the manuscript for important intellectual content. MV assisted with the collection of data and analysis and interpretation of data.

## Disclaimer

 The views expressed in this paper represent the opinions of the authors and not an official position of the institutions of their affiliation.

## Authors’ affiliations

^1^Health Policy PhD Program, McMaster University, Hamilton, ON, Canada. ^2^McMaster Health Forum, McMaster University, Hamilton, ON, Canada. ^3^Department of Health Evidence and Impact, McMaster University, Hamilton, ON, Canada. ^4^Centre for Health Economics and Policy Analysis, McMaster University, Hamilton, ON, Canada. ^5^Department of Political Science, McMaster University, Hamilton, ON, Canada. ^6^Africa Centre for Evidence, University of Johannesburg, Johannesburg, South Africa. ^7^Knowledge to Policy Center, American University of Beirut, Beirut, Lebanon. ^8^Universidad de Antioquia, Medellin, Colombia.

## Supplementary files


Supplementary file 1. The Search Strategy for Four Databases.
