# Supplementary material for: Examining and Contextualizing Approaches to Establish Policy Support Organizations – A Critical Interpretive Synthesis
Source: Int J Health Policy Manag. 2020 Sep 30;11(5):551–66. doi: 10.34172/ijhpm.2020.181 (PMC9309922; doi:10.34172/ijhpm.2020.181)
Supplement: Supplementary file 1 — The Search Strategy for Four Databases. [file ijhpm-11-551-s001.pdf]

Supplementary file 1. The Search Strategy for Four Databases

Medline, Healthstare, Embase, PsycINFO

Database: OVID Medline Epub Ahead of Print, In-Process & Other Non-Indexed Citations, Ovid MEDLINE(R) Daily and Ovid MEDLINE(R) 1946 to Present

Search Strategy:

----

1 (KT adj2 (translat* or support or inform or based or implement* or analysis or formulat* or develop* or disseminat* or utili*or application or synthes* or transfer or exchange or diffus* or uptake)).ti,ab. (518)

2 (knowledge adj2 (translat* or support or inform or based or implement* or analysis or formulat* or develop* or disseminat* or utili*or application or synthes* or transfer or exchange or diffus* or uptake)).ti,ab. (22384)

3 (evidence* adj2 (translat* or support or inform or based or implement* or analysis or formulat* or develop* or disseminat* or utili*or application or synthes* or transfer or exchange or diffus* or uptake)).ti,ab. (145986)

4 (research* adj2 (translat* or support or inform or based or implement* or analysis or formulat* or develop* or disseminat* or utili*or application or synthes* or transfer or exchange or diffus* or uptake)).ti,ab. (73072)

5 polic*.ti,ab. (233636)

6 exp Policy Making/ (23629)

7 Decision Making/ (85010)

8 policy mak*.ti,ab. (22273)

9 policy-mak*.ti,ab. (22273)

10 decision mak*.ti,ab. (117071)

11 decision-mak*.ti,ab. (117071)

12 decisionmak*.ti,ab. (1049)

13 (institut* or centre or center or platform* or committee or unit or observatory or directorate or forum or council or Think tank* or Thinktank*).ti,ab. (1382591)

14 health.ti,ab. (1585698)

15 medical.ti,ab. (1019646)

16 policymak*.ti,ab. (9849)

17 1 or 2 or 3 or 4 (232296)

18 5 or 6 or 7 or 8 or 9 or 10 or 11 or 12 or 16 (405188)

19 14 or 15 (2387866)

20 13 and 17 and 18 (6035)

21 13 and 17 and 18 and 19 (4469)

22 limit 21 to English language (4273)
